# Obesity during Adolescence and Feeding Practices during Infancy: Cross-Sectional Study

**DOI:** 10.3390/epidemiologia4010011

**Published:** 2023-03-21

**Authors:** Reem Sharaf-Alddin, Radhia Almathkoori, Hara Kostakis, Ahmed N. Albatineh, Abdullah Al-Taiar, Muge Akpinar-Elci

**Affiliations:** 1 CONRAD, Eastern Virginia Medical School, Norfolk, VA 23507, USA; 2 Department of Public Health, Ministry of Health, Kuwait City 13001, Kuwait; 3 London School of Hygiene & Tropical Medicine, University of London, London WC1E 7HT, UK; 4 Faculty of Medicine, Kuwait University, Kuwait City 13110, Kuwait; 5 School of Community & Environmental Health, College of Health Sciences, Old Dominion University, Norfolk, VA 23508, USA; 6 School of Public Health, University of Nevada, Reno, NV 89557, USA

**Keywords:** breastfeeding, infancy, obesity, adolescence

## Abstract

Background: Breastfeeding is proposed to play a role in reducing the risk of obesity throughout life. Kuwait has an extremely high prevalence of childhood obesity (45% of adolescents are overweight/obese) and extremely low breastfeeding indicators, particularly exclusive breastfeeding. In fact, little is known about the association between breastfeeding and obesity from Kuwait and the broader Middle East. Aims: To estimate the prevalence of overweight/obesity in female adolescents in Kuwait and assess its association with breastfeeding during infancy. Methods: This is a cross-sectional study that included 775 girls randomly selected from public and private high schools in Kuwait. The primary exposure was breastfeeding in the first four months of life, and the outcome was overweight/obesity during adolescence. Multivariable logistic regression was used to assess the association between breastfeeding and overweight/obesity while adjusting for potential confounders. Results: Approximately 45% of adolescent girls were either overweight/obese. We found no significant association between breastfeeding (exclusive/mixed breastfeeding and formula feeding/no breastfeeding) and overweight/obesity neither in univariable analysis (Crude Prevalence Ratio: 1.14, 95%CI [0.92–1.36] & Crude Prevalence Ratio: 1.29, 95%CI [0.86–1.68]; *p* = 0.293) for mixed feeding and no breastfeeding respectively, nor in multivariable analysis (Adjusted Prevalence Ratio: 1.14, 95%CI [0.85–1.42] & Adjusted Prevalence Ratio: 1.20, 95%CI [0.68–1.68]; *p* = 0.589) for mixed feeding and no breastfeeding respectively. Conclusion: Breastfeeding during infancy was not significantly associated with overweight/obesity during adolescence. However, breastfeeding should be encouraged for its indisputable benefits for infants and their mothers alike. Further prospective studies are needed to assess the association.

## 1. Background

The benefit of breastfeeding for both mothers and their infants are now well-recognized beyond any doubt. Breastfed infants, compared to formula-fed infants, have better neurological development [1], stronger immune systems, and a lower rate of hospitalization [2]. Despite these obvious short-term benefits, breastfeeding practices remained not optimal worldwide. Globally, only 41% of infants are exclusively breastfed during the first six months of life [3]. Kuwait has lower breastfeeding indicators compared to many low, middle, and high-income countries [4]. Only 10.15% and 8.41% of infants aged ≤3 and ≤6 months are exclusively breastfed, respectively, and around 8 out of 10 Kuwaiti infants aged ≤6 months are fed formula milk [5].

Childhood obesity and its consequences represent a major public health problem worldwide. Globally, about 18% of those aged 5–19 years are overweight/obese [6]. In Kuwait, almost half of school children aged 5–19 years are overweight/obese [5]. As a result, Kuwait is among the top ten countries in terms of obesity [7], and type II diabetes is the number one health problem in Kuwait (14.6% of Kuwaiti adults are diabetic) [8].

According to Barker hypothesis, later known as Developmental Origin of Health and Disease (DOHaD), exposure at early life (during pregnancy or early childhood) is assumed to have a long-term impact on health [9]. Breastfeeding, as an early life exposure, has been proposed to play a role in reducing the risk of overweight/obesity throughout life. One of the explanations is that breastmilk contains leptin, a hormone that works to ensure energy balance and control metabolic processes [10]. Leptin stimulates lipid catabolism and inhibits lipogenesis, hence controlling fat accumulation from an early stage of life, and consequently lowers the risk of obesity-related health problems in adulthood [11].

Several epidemiological studies have attempted to demonstrate the link between breastfeeding during infancy and the risk of overweight/obesity in adolescence or adulthood, but the findings remained inconclusive [12]. Several studies have shown a negative association, with some demonstrated a dose-response relationship [13]. On the contrary, other studies reported little or no association between breastfeeding during infancy and overweight/obesity throughout life [14].

As mentioned above, Kuwait has an extremely high prevalence of childhood obesity [5] and extremely low breastfeeding indicators, particularly exclusive breastfeeding [5]. Therefore, investigating the association between breastfeeding in infancy and obesity or overweight in adolescence is of utmost importance in our setting. If this association is demonstrated in our setting, it will revive the effort to improve breastfeeding as an early intervention to combat childhood obesity. In fact, little is known about this issue from Kuwait and the broader Middle East.

### Objectives

This study aims to investigate the association between breastfeeding during infancy and overweight/obesity during adolescence.

## 2. Methods

### 2.1. Study Design and Study Participants

Kuwait has a population of 4.5 million, of whom two-thirds are non-Kuwaiti. Education is compulsory until secondary school for Kuwaiti, and school enrolment is very high for both genders. Only less than 1% of Kuwaiti females aged 15–18 years, which is the study’s target population, are illiterate [15]. The government partially subsidizes formula milk, and there is only one private and one public baby-friendly hospital in Kuwait.

**Figure 1:**
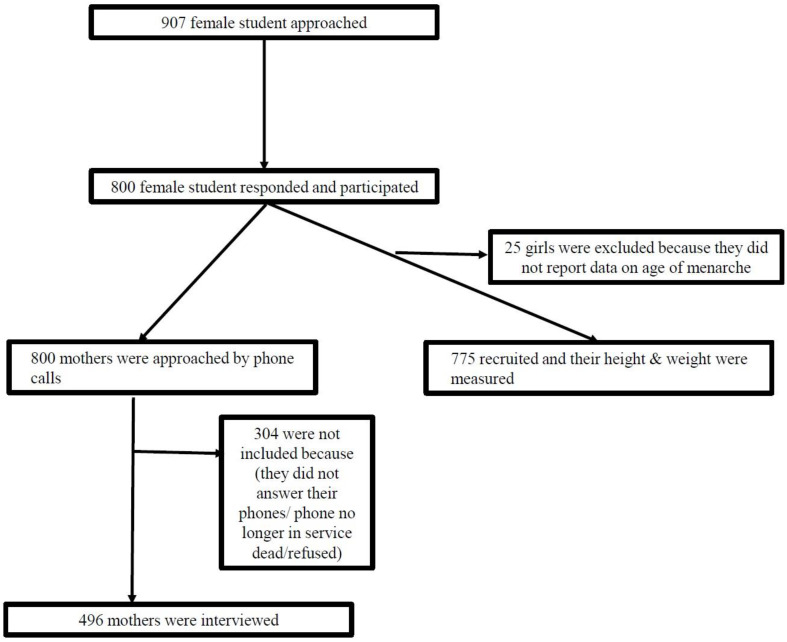
Study profile.

This is a cross-sectional study in which data were collected on schoolgirls attending public and private high schools in Kuwait (age range: 14–22 years). The data for this study is part of a project, and details of the project have been published previously [16,17]. The project profile is depicted in Figure 1.

### 2.2. Data Collection

Data collection has been described in detail previously [16,17]. In brief, data were collected from schoolgirls by a self-administered questionnaire, while data from mothers were collected through telephone interviews using a structured questionnaire. Mothers were the only recognized source of information about the history of breastfeeding. The telephone interview with the mothers were conducted independently from the time of measuring the schoolgirls height and weight. Which means the researcher, during the phone interview with the mother was not aware about the adolescent girl (the daughter) BMI status. This is to avoid observer bias that might arise when the data collector is aware about the study hypothesis, hence overestimate the association. In order to improve the response rate, those mothers who did not respond at the first time were approached three times at different timing through their cell phones and landline.

Breastfeeding was defined as infant was fed with breastmilk either directly from the mother’s breast or by a cup or a bottle [18]. Questions on breastfeeding were rephrased to aid recall. Questions were focused on the feeding practices during the first four months of life. This included whether the schoolgirl was breastfed at all, and whether it was exclusive (Breastmilk only) or mixed feeding (Breastmilk with formula milk/solid food). Further questions aimed to ascertain the exact duration of breastfeeding among mothers who breastfed. Those who were not able to recall the information in any question were given a code and considered as a category during the analysis.

Height and weight were measured in the school theatre or school clinic for privacy. Weight was measured by the school nurse using a digital weight scale (Beurer GS 19 digital scale, Ulm, Germany) and recorded to the nearest 0.1 kg after removing heavy clothes and shoes. Height was measured using a stadiometer (Seca 217 height rod, Hamburg, Germany) and recorded to the nearest 0.1 cm after the participant was correctly positioned and her position was verified by the researcher from the front and left sides.

A set of potential confounders was selected based on previous knowledge about the factors that might be associated with obesity including sociodemographic factors, dietary factors, and based on Barker hypothesis (including complication during pregnancy, birth weight and prematurity). Data on potential confounders were collected by self-administered questionnaires from the schoolgirls and telephone interviews from the mothers. The questionnaires included data on sociodemographic factors, index girls’ (age, nationality, birth order, birth weight, whether born premature or not, exposed to passive smoking at home, age of menarche), age of the mother at index girls’ birth and whether she experienced complications (e.g., eclampsia or gestational diabetes) during that pregnancy (Table 1).

Data on lifestyle factors (dietary habits, physical activity, and physical inactivity) were collected by questions that have been used previously among adolescents in Arab settings. Dietary habits including frequency per week of (eating vegetables, fresh fruits, fast food, fries/chips, cake/biscuit/donuts, sweats/chocolate, having soft drinks). Physical activity factors including, the frequency and duration per week of (attending PE in school, walk to school, biking, walking, jogging, running, swimming, play football, play basketball, play volleyball, practice self-defense sports, do weight training and body building). Physical inactivity including, the frequency and duration per week of (watching TV, using internet, playing video games, reading book, doing homework). The questionnaires for mothers and daughters were developed in English and translated into Arabic, then independently back-translated into English. The Arabic versions of the questionnaires were pilot tested on 30 mothers and schoolgirls whose data were not included in the study.

### 2.3. Data Management and Data Analysis

Body mass index (BMI) was calculated by dividing the weight in Kg by the square of height in meter (Kg/m^2^). BMI-for-age z-scores were calculated using WHO growth charts. Overweight was defined as BMI-for-age greater than one standard deviation (SD) to two SD; while obesity as greater than 2 SD using the WHO growth reference median [19]. We found few schoolgirls older than 18 years; therefore, we used BMI cut-off points for adults for those girls (<18.5 kg/m^2^ underweight, 18.5–24.9 kg/m^2^ normal weight, 25.0–29.9 kg/m^2^ overweight, >30.0 kg/m^2^ obese) [19].

Continuous variables were summarized by mean (SD) (if were found normally distributed) and categorical variable were summarized by percentages and frequencies. In order to investigate the association between breastfeeding and overweight/obesity, we created a binary outcome (underweight/normal weight vs. overweight/obese). Only 12 participants were underweight; hence they were included in the normal weight. Chi-square test for independence was used to assess the differences in overweight/obesity prevalence between those who were exclusively breastfed, mixed feeding (breastmilk and formula-milk), or never breastfed. Since our outcome is binary variable (Overweight/obesity vs. Normal weight), We used binary logistic regression to investigate the association between breastfeeding and overweight/obesity. Logistic Regression calculates the Odds Ratio (OR) with its 95% CI of the association while adjusting for multiple covariates. The goodness of Model fit was assessed by Hosmer-Lemeshow test. Because overweight/obesity was common (rare disease assumption was not met), we calculated prevalence ratio (PR) instead of odds ratio (OR) using Stata command “*oddsrisk*,” as described by Zhang et al. [20]. Separate analyses were used for the following infants’ feeding practices: breastfeeding (ever breastfed/never breastfed), breastfeeding type (exclusive/mixed/no breastfeeding), duration of breastfeeding (as ≤4 vs. >4 months & or ≤6 vs. >6 months), formula-milk (yes/no), age of introducing formula-milk (≤4 vs. >4 months), and age of introducing solid food (as ≤4 months vs. >4 months & ≤6 vs. >6 months). First, crude PR was calculated for breastfeeding and each of the infants’ feeding practices as well as other covariates. Then, adjusted PR was calculated by introducing covariates that showed association at 20% level of significance in univariable analysis. The impact of this on the association was noted by comparing the crude and adjusted PR. The steps of the univariable and multivariable analyses were repeated for each of the infants’ feeding practices.

## 3. Results

Of the 907 students selected, 800 (88.2%) responded, and 775 (85.4%) were included in this analysis (Figure 1). The mean (SD) age of the study participants was 16.7 (1.1) years. About three-quarters of the schoolgirls were Kuwaiti (76.8%), and most of them were from public schools (77.8%). The prevalence of overweight or obesity was (23.6%) and (22.2%) respectively, Table 2. This was not significantly different between public and private schools (*p* = 0.926) nor between Kuwaiti and non-Kuwaiti nationals (*p* = 0.853).

Table 3 shows factors that were significantly associated with overweight/obesity in univariable analysis. These factors were age of the schoolgirls, their living arrangement (whether living with both parents or not), mother’s level of education, number of offspring’s the mother has, age of menarche of the schoolgirls, and the frequency of weekly consumption of each (soft drinks, fruits, dairy products and fries).

Of 800 mothers approached by telephone calls, 496 responded; hence the association between overweight/obesity and infants’ feeding practices before and after adjusting for the potential confounders shown in Table 4 is for 496 schoolgirls. Whether the participant was ever breastfed or not showed no association with overweight/obesity; crude PR = 1.32 [95%CI: 0.81–1.74], (*p* = 0.214) and adjusted PR = 1.07 [95%CI: 0.52–1.65], (*p* = 0.813). Similarly, type of breastfeeding (exclusive, mixed, no breastfeeding) during the first four months of life was not significantly associated with overweight/obesity in univariable (*p* = 0.293) or multivariable analyses (*p* = 0.589). There was no significant association between breastfeeding duration and overweight/obesity, whether it was fitted as a continuous or a categorical variable. We categorized breastfeeding duration as (≤4 months and >4 months) of birth and as (≤6 months and >6 months) and conducted separate analyses. In both analyses, no significant association was found between duration of breastfeeding and overweight/obesity.

Whether the participant was formula fed or not was not significantly associated with overweight/obesity in both univariable (*p* = 0.410), or multivariable analysis (*p* = 0.758). There was no significant association between age at which solid food was introduced and overweight/obesity whether age was fitted as a continuous variable (*p* = 0.354), or categorized as (≤4 vs. >4 months) of life (*p* = 0.643). However, when we re-categorized this variable as (≤6 months and >6 months) of life, there was a significant association with overweight/obesity in both univariable and multivariable analysis; crude PR: 1.42 [95%CI: 1.13–1.68], (*p* = 0.019) and adjusted PR:1.77 [95%CI: 1.39–2.03], (*p* < 0.001).

The other factors that showed significant association with overweight/obesity in multivariable analysis were age of menarche (*p* = 0.039), weekly consumption of dairy products (*p* = 0.008), and weekly consumption of fries (*p* = 0.040).

We also conducted separate analyses while re-categorizing BMI as overweight, normal weight in one category, and obese in the other category. The analysis revealed the same conclusion- i.e., no association between breastfeeding or breastfeeding duration during infancy and obesity during adolescents. Additionally, the analysis was repeated while excluding the 12 participants who were underweight and the result remained unchanged. Finally, we used stepwise selection to identify factors associated with overweight/obesity using stepwise logistic regression and none of the variables related to breastfeeding or infants’ feeding was selected in this method; hence our conclusion from the main analysis above remained practically unchanged.

## 4. Discussion

This study aimed to examine the association between overweight/obesity and feeding practices during infancy. We estimated the prevalence of overweight and obesity to be (23.61%) and (22.19%) respectively and found no significant association between breastfeeding during infancy and overweight/obesity during adolescence.

The estimated prevalence of overweight and obesity is similar to that reported by Kuwait Nutritional Surveillance System (47.13% of schoolgirls aged 15–19 years were overweight/obese) [5]. It is also similar to that reported in many other studies in Kuwait [21] and neighboring countries. For example, the prevalence of overweight or obesity among schoolgirls was (40.4%) in Qatar [22], and (41.4%) in United Arab Emirates [23]. Efforts should be made to combat overweight and obesity in adolescents in Kuwait in order to reduce its short-term and long-term impact on the health of the population.

The prevalence of overweight/obesity among the 496 participants who have their breastfeeding status reported by their mothers is about 46% which is the same as that of the total number of participants including those who we did not have their breastfeeding status reported.

Among the 496 participants who have their breastfeeding status reported by their mothers, we found no association between breastfeeding (exclusive or mixed feeding) in the first four months of life and overweight/obesity during adolescence in female schoolgirls in Kuwait. Also, we found no association between breastfeeding duration in infancy and overweight/obesity during adolescence in female schoolgirls (Table 4). Our findings are consistent with other studies that showed no association between breastfeeding during infancy and obesity during childhood or adolescence, including longitudinal studies [14,24]. However, other studies suggested a protective effect or an inverse association between breastfeeding during infancy and overweight/obesity later in life. For instance, A meta-analysis included 25 studies published between 1997 to 2014 revealed that there is a protective effect of breastfeeding against obesity during childhood, with 17 out of 25 studies found a dose-response relationship, although the authors acknowledged the role of publication bias in their findings [13].

Although it is possible that there is no association between breastfeeding during infancy and overweight/obesity and that our findings truly describe the reality, there are other possible explanations for the lack of association in our study. Collecting data on breastfeeding and breastfeeding duration with a long recall period may have resulted in non-differential misclassification that attenuated the association towards the null. The other possible explanation for the lack of association between overweight/obesity and breastfeeding in our study could be the confounding by diet. Although we collected data on the current diet, diet may have changed over time from the time our study participants were infants to the time they became adolescents. Furthermore, collecting data on diet using food frequency questionnaire always results in a substantial non-differential misclassification, which in our case would results in residual confounding, another explanation for our negative findings. Another explanation could be that we have only 18 participant who never been breastfed during infancy, which would impact the findings since we will not have enough power to detect the association. However, we conducted the analysis based on the duration of breastfeeding with numbers of participants in the categories being large enough to detect meaningful association and our findings remain the same in both analyses. Second, the data about breastfeeding was available for 496 (64% of the total number of participants). If those we do not have their breastfeeding status are different from the 496 with reported breastfeeding this might impact the association. However, we can see that the prevalence of obesity/overweight among the 496 is (46%) which is similar to the prevalence in the whole study group (45.8%). This is reassuring as those who completed the interview are not different from those who did not in terms of overweight/obesity. Finally, it is possible that our negative findings could be due to confounding by genetic or epigenetic factors, which we did not measure in our study.

We also found no association between whether schoolgirls were formula-fed during infancy and overweight/obesity during adolescence. Our findings are different from that reported in other studies that suggest a positive association between formula-milk intake and weight gain or overweight/obesity [25]. We also found no association between the age at which formula milk was introduced and overweight/obesity, which is different from other studies that reported negative association (i.e., early initiation of formula milk associated with a higher risk of overweight/obesity) [26]. The absence of association in our analysis could be due to the fact that most of the participants consumed formula milk. Similar to the above, the lack of association could be due to non-differential misclassification due to a long recall period or confounding by other factors such as diet, genetic or epigenetic factors.

We found no association between the age at which solid food was introduced and overweight/obesity (≤4 moths vs. >4 months of life). This is consistent with studies from other settings among breastfed children there was no association between early introduction of solid food (<4 months of life) and prevalence of obesity [27,28]. In contrast, in a systematic review, the early introduction of solid food at ≤4 months was associated with an increased risk of childhood overweight [28]. Another study found that [27].

Although we found no association between overweight/obesity and age of introducing solid food when categorized as (≤4 moths vs. >4 months) which similar to previous studies [28], we found a significant positive association between age of introducing solid food when categorized into (at >6 months). Such unexpected finding has been reported previously from other studies that found late introduction of solid food (at ≥9 months) was positively associated with obesity during childhood [28,29].

In our analysis, there are several explanations for this association. First, 67 mothers (15.69% of the participants) did not remember the age at which they introduced solid food. Our findings may change if these mothers remembered the age at which they introduced solid food for their daughters. Second, studies suggested that it is the quality of solid food that is more influential on BMI or overweight/obesity rather than the time of introducing solid food [30,31]. No data were collected on the type of solid food introduced in our study; and such data would be unreliable with this long recall period. Third, some studies have suggested that the interaction between breastfeeding and the age at which solid foods were introduced, is related to obesity later at life [27]. Therefore, we assessed for interaction in our analysis and found no interaction.

This is the first study that assessed the association between breastfeeding during infancy and overweight/obesity during adolescence among females in Kuwait and Gulf region, where obesity is a major health problem in this age group. This study was conducted among nationally representative sample of adolescent females. The participants were recruited from both public and private schools and in Kuwait almost all girls in this age group are enrolled in schools. However, there are several limitations in our study that require consideration while interpreting the data. First, data on breastfeeding (the exposure) were dependent on mother’s ability to recall their practices over a long time period. Although the questions were designed to aid accurate recall, there is possibility for non-differential misclassification in breastfeeding, which attenuates the association between breastfeeding during infancy and overweight/obesity during adolescence. Second, the association could be confounded by factors that we did not address in this analysis (e.g., genetic or epigenetic factors). Third, using BMI as a proxy for obesity (fat accumulation) is not accurate to determine adiposity since it might reflect greater bone density or muscle bulk but not necessarily excess body fat. This might lead to non-differential misclassification of the outcome, which attenuates the association towards the null.

## 5. Conclusions

In conclusion, we found no significant association between breastfeeding or breastfeeding duration during infancy and overweight/obesity during adolescence. Breastfeeding has other indisputable benefits for mothers and children and should be encouraged whether or not it is associated with obesity later at life. Further longitudinal studies that collect data on breastfeeding and other feeding practices prospectively from birth until adolescence are needed to elucidate the long-term benefits of breastfeeding in terms of obesity during adolescence. Such studies should collect data on potential confounders such as genetic and epigenetic factors in addition to repeatedly monitor diet over the whole study period.

## Figures and Tables

**Table 1:** Sociodemographic characteristics of 775 Kuwaiti and non-Kuwaiti adolescent girls in Kuwait.

Characteristics	Public (n = 603)		Private (n = 172)		Total (N = 775)	
	n	(%)	n	(%)	N	(%)
**Age in Years, Mean (SD)**	16.74	(1.09)	16.62	(1.21)	16.72	(1.12)
						
**Nationality ^1^**						
Kuwaiti	572	(95.33)	21	(12.21)	593	(76.81)
Non-Kuwaiti	28	(4.67)	151	(87.79)	179	(23.19)
						
**Father’s Education ^2^**						
No formal education	4	(0.72)	6	(3.77)	10	(1.40)
Primary/Intermediate	60	(10.81)	27	(16.98)	87	(12.18)
Secondary (high school)	143	(25.77)	34	(21.38)	177	(24.79)
Diploma	81	(14.59)	15	(9.43)	96	(13.45)
University & above	267	(48.11)	77	(48.43)	344	(48.18)
						
**Mother’s Education ^3^**						
No formal education	23	(3.97)	14	(8.48)	37	(4.97)
Primary/Intermediate	74	(12.76)	36	(21.82)	110	(14.77)
Secondary (high school)	141	(24.31)	33	(20.00)	174	(23.36)
Diploma	81	(13.97)	15	(9.09)	96	(12.89)
University & above	261	(45.00)	67	(40.61)	328	(44.03)
						
**Currently residing with ^4^:**						
Both parents	520	(86.52)	156	(91.23)	676	(87.56)
Mother alone	67	(11.15)	9	(5.26)	76	(9.84)
Father alone	5	(0.83)	2	(1.17)	7	(0.91)
Other family members	9	(1.50)	4	(2.34)	13	(1.68)

^1^ Missing for 3 participants; ^2^ Missing for 61 participants; ^3^ Missing for 30 participants; ^4^ Missing for 3 participants.

**Table 2:** Prevalence of overweight or Obesity in 775 female students in public and private schools in Kuwait.

BMI Characteristics	Public (n = 603)		Private (n = 172)		Total (N = 775)	
	n	(%)	n	(%)	N	(%)
						
**Underweight**	9	(1.49)	3	(1.74)	12	(1.55)
**Normal weight**	314	(52.07)	94	(54.65)	408	(52.65)
**Overweight**	144	(23.88)	39	(22.67)	183	(23.61)
**Obese**	136	(22.55)	36	(20.93)	172	(22.19)
**Total**	593	(100.0)	179	(100.0)	772	(100.0)

**Table 3:** Factors associated with overweight/obesity among 775 females in Kuwait in univariable analysis.

Characteristics	Total	Prevalence of Overweight/Obesity		PR	[95%CI]	*p* ^a^
		N	(%)			
**Sociodemographic factors**						
**Age in years**						
14–16	258	127	(49.22)		[Reference]	0.048
>16–17	257	125	(48.64)	0.99	[0.80–1.18]	
>17	260	103	(39.62)	0.79	[0.62–0.98]	
**Currently residing with:**						
Both parents	676	320	(47.34)	1	[Reference]	0.017
Not with both parents ^b^	96	33	(34.38)	0.73	[0.53–0.91]	
**‘Mothers’ highest level of education**						
University & above	328	158	(48.17)	1	[Reference]	0.037
Diploma	96	45	(46.88)	0.97	[0.73–1.22]	
Secondary (high school)	174	86	(49.43)	1.03	[0.83–1.23]	
Primary or intermediate school	110	41	(37.27)	0.76	[0.56–0.99]	
No formal education	37	10	(27.03)	0.55	[0.30–0.91]	
**Age of menarche in years**						
7–<12 years	201	111	(55.22)	1	[Reference]	0.003
12–<13 years	209	95	(45.45)	0.80	[0.61–0.99]	
≥13 years	231	90	(38.96)	0.67	[0.50–0.86]	
**Number of children the mother has**						
1–3 children	83	43	(51.81)	1	[Reference]	0.003
4–5 children	198	106	(53.54)	1.04	[0.75–1.35]	
6 children or more	214	81	(37.85)	0.69	[0.47–0.97]	
**Nutritional habits**						
**Weekly consumption of soft drinks**						
≤3 times per week	490	239	(48.78)	1	[Reference]	0.026
>3 times per week	277	112	(40.43)	0.83	[0.69–0.98]	
**Weekly consumption of fruits**						
≤3 times per week	509	215	(42.24)	1	[Reference]	0.008
>3 times per week	262	137	(52.29)	1.24	[1.06–1.41]	
**Weekly consumption of dairy products**						
≤3 times per week	341	130	(38.12)	1	[Reference]	<0.001
>3 times per week	429	221	(51.52)	1.35	[1.16–1.54]	
**Weekly consumption of fries**						
≤3 times per week	490	239	(48.78)	1	[Reference]	0.026
>3 times per week	284	115	(40.49)	0.83	[0.69–0.80]	

^a^ *p*-value calculated by chi-squared test for independence, ^b^ Either with the father without the mother, or with the mother without the father, or with a family member other than the parents.

**Table 4:** Association between overweight/obesity and ‘ ‘infant’s feeding practices in the first four months of life before and after adjusting for potential confounders.

Characteristics	Total	Prevalence of Overweight/Obesity		Model I			Model II		
				PR	[95%CI]	*p* ^a^	PR	[95%CI]	*p* ^b^
	N	(%)							
**Have you ever breastfed your daughter?**									
**Yes**	476	220	(46.22)	1	[Reference]	0.214	1	[Reference]	0.813
No	18	11	(61.11)	1.32	[0.81–1.74]		1.07	[0.52–1.65]	
**Type of ‘infants’ feeding in the first 4 months**									
Breastfeeding only	150	63	(42.00)	1	[Reference]	0.293	1	[Reference]	0.589
Breastfeeding & formula milk	313	151	(48.24)	1.14	[0.92–1.36]		1.14	[0.85–1.42]	
No Breastfeeding	31	17	(54.84)	1.29	[0.86–1.68]		1.20	[0.68–1.68]	
**The duration of breastfeeding exclusive/mixed feeding (>6 months)**									
≤6 months	425	200	(47.06)	1	[Reference]	0.954	1	[Reference]	0.410
>6 months	59	28	(47.46)	1.01	[0.73–1.29]		0.84	[0.52–1.22]	
**The duration of breastfeeding exclusive/mixed feeding (>4 months)**									
≤4 months of age	330	158	(47.88)	1	[Reference]	0.619	1	[Reference]	0.381
>4 months	154	70	(45.45)	0.95	[0.76–1.15]		0.88	[0.64–1.15]	
**Have you ever fed your daughter with formula milk?**									
Yes	425	202	(47.53)	1	[Reference]	0.410	1	[Reference]	0.758
No	71	30	(42.25)	0.89	[0.64–1.15]		1.06	[0.70–1.43]	
**Age of introducing formula milk (in months) ^**									
≤4 months of age	335	163	(48.66)	1	[Reference]	0.702	1	[Reference]	0.214
>4 months	63	29	(46.03)	0.95	[0.68–1.22]		0.77	[0.46–1.14]	
**Age of introducing solid food (≤4 vs. >4 months)**									
≤4 months of age	147	70	(47.62)	1	[Reference]	0.948	1	[Reference]	0.643
>4 months	270	127	(47.04)	0.99	[0.78–1.20]		0.97	[0.72–1.24]	
‘Don’t remember	67	33	(49.25)	1.03	[0.74–1.33]		1.15	[0.77–1.50]	
**Age of introducing solid food (≤6 vs. >6 months)**									
≤6 months of age	354	157	(44.35)	1	[Reference]	0.019	1	[Reference]	<0.001
>6 months	63	40	(63.49)	1.42	[1.13–1.68]		1.77	[1.39–2.03]	
‘Don’t remember	67	33	(49.25)	1.11	[0.83–1.39]		1.31	[0.91–1.67]	

^a^: *p*-value calculated by chi-squared test for independence; ^b^: *p*-value calculated by likelihood ratio test; ^ excluded those who ‘don’t remember. Model I: unadjusted; Model II: adjusted for age (categorical) of the student, living arrangement (student living with both parents or not), ‘mothers’ education, family monthly income, ‘participants’ age of menarche(categorical), number of children the mother has, ‘mothers’ age in her first pregnancy(categorical), whether the mother had medical problems during the pregnancy of the participant girl, weekly consumption of (soft drinks, vegetables, fruits, dairy products, fries, cakes or biscuits, sweets or chocolates), hours of (walking for exercising, and using internet) per week.

## Data Availability

This is a secondary data analysis.
